# Rescue computed tomography-guided drainage of a giant mediastinal mature teratoma causing airway obstruction prior to surgical resection: a case report

**DOI:** 10.1186/s40792-023-01638-3

**Published:** 2023-04-13

**Authors:** Kengo Tani, Daisuke Kimura, Tsubasa Matsuo, Yoshiaki Saito, Kageaki Taima, Shinya Kakehata, Akira Kurose, Masahito Minakawa

**Affiliations:** 1 Department of Thoracic and Cardiovascular Surgery, Hirosaki University Graduate School of Medicine, 5 Zaifu-cho, Aomori 036-8562 Hirosaki, Japan; 2 Department of Respiratory Medicine, Hirosaki University Graduate School of Medicine, 5 Zaifu-cho, Aomori 036-8562 Hirosaki, Japan; 3 Department of Diagnostic Radiology, Hirosaki University Graduate School of Medicine, 5 Zaifu-cho, Aomori 036-8562 Hirosaki, Japan; 4 Department of Anatomic Pathology, Hirosaki University Graduate School of Medicine, 5 Zaifu-cho, Aomori 036-8562 Hirosaki, Japan

**Keywords:** CT-guided drainage, Mediastinal mature teratoma, Giant mediastinal tumor, Airway obstruction

## Abstract

**Background:**

Giant mediastinal mature teratomas may cause airway obstruction or decreased venous return due to the mass effect. Preoperative stabilization of the respiratory and circulatory systems is important for perioperative management to safely perform surgery, including general anesthesia. However, to the best of our knowledge, there are only a few reports regarding the preoperative computed tomography (CT)-guided drainage of mediastinal tumors.

**Case presentation:**

A 30-year-old woman was admitted to the emergency room with sudden dyspnea. CT findings revealed a giant cystic mass in the anterior mediastinum compressing the trachea and the right main bronchus. The patient was intubated and CT-guided drainage of the fluid content of the cyst was performed to decompress the airway obstruction. Thereafter, the mediastinal tumor was resected during elective surgery and pathologically diagnosed as a mature teratoma.

**Conclusions:**

Rescue preoperative CT-guided drainage of a giant mediastinal mature teratoma allowed safe general anesthesia and surgery by releasing the airway obstruction.

## Background

A mediastinal mature teratoma is a benign tumor. However, a large tumor can cause respiratory and circulatory failure due to the compression of the surrounding organs. In cases of respiratory or circulatory collapse, extracorporeal life support (ECLS) is required for multidisciplinary treatment of giant teratomas. We encountered a case of a giant mediastinal mature teratoma, in which computed tomography (CT)-guided drainage was effective in relieving the airway obstruction prior to surgical resection. To our knowledge, there are few reports of preoperative CT-guided drainage for giant mediastinal mature teratomas. Therefore, we report this case and discuss the availability of preoperative CT-guided drainage for giant mediastinal mature teratomas.

## Case presentation

**Fig. 1:**
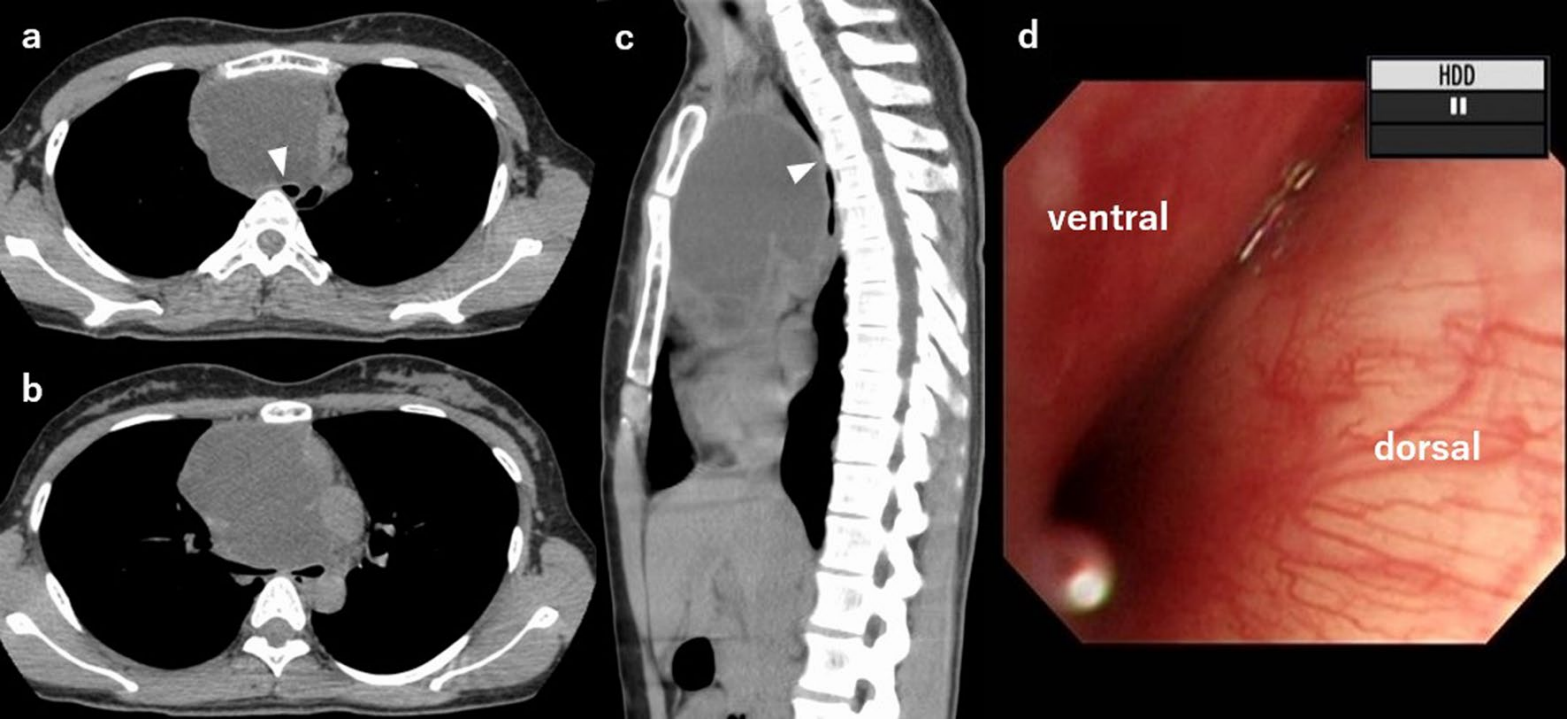
CT and bronchoscopy findings before drainage. **a** CT image at the trachea level. A giant anterior mediastinal cystic mass compressing the trachea was noted. The trachea was deviated to the left (white arrowhead). **b** CT image at the level of carina of the trachea. **c** Sagittal section of the CT image. The trachea was compressed (white arrowhead). **d** Bronchoscopy image at the trachea level. The trachea was compressed and it was difficult to observe the right main bronchial inlet. *CT* computed tomography

A 30-year-old female patient had been experiencing anterior chest pain and cough for the past 6 months. CT scan by the primary care doctor revealed a giant anterior mediastinal polycystic tumor (Fig. 1a–c). The tumor was 11.2 × 7.7 × 7.2 cm in size and compressed the trachea and the right main bronchus. A thymoma or germ-cell tumor was suspected as the differential diagnosis. The patient was referred to our hospital, but before visiting the outpatient clinic, she suddenly experienced dyspnea and came to the emergency room. Her blood pressure was 154/84 mmHg and her heart rate was indicative of sinus tachycardia, at 135/min. Her respiratory rate was 16/min, and her saturation of percutaneous oxygen (SpO_2_) was 94% on room air. The arterial blood gas on room air showed a partial pressure of oxygen (PaO_2_) of 72 mmHg and a partial pressure of carbon dioxide (PaCO_2_) of 33 mmHg. Wheezing was observed in her lung auscultation, and she was unable to remain in a supine position owing to respiratory distress. Blood laboratory data showed normal levels of alpha-fetoprotein, human chorionic gonadotropin, soluble interleukin-2 receptor, white blood cells (7950/µL), and C-reactive protein (0.03 mg/dL). The level of anti-acetylcholine receptor antibody was not evaluated. The CT findings indicated that airway stenosis might progress and the patient’s general condition was expected to worsen; thus, we decided to administer endotracheal intubation.

**Fig. 2:**
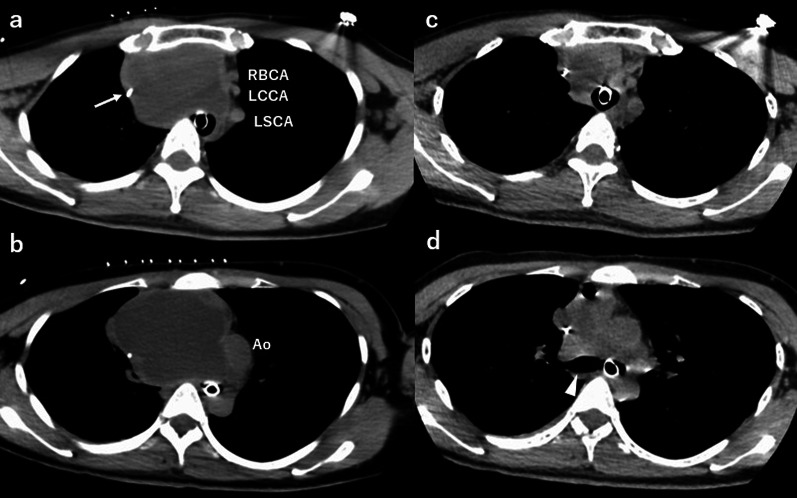
Comparison of CT images before and after drainage. A double-lumen tube was intubated into the left main bronchus. Central venous catheter was inserted from the right internal jugular vein (white arrow). The images **a** and **b** were taken before drainage. The images **c** and **d** were taken after drainage. The images **a** and **c** are at the trachea level and images **b** and **d** are at the level of carina of the trachea. After drainage, the right main bronchus was patent (white arrowhead). *CT* computed tomography, *RBCA* right brachiocephalic artery, *LCCA* left common carotid artery, *LSCA* left subclavian artery, *Ao* aorta

She was intubated by an endotracheal tube, but manual ventilation was poor. The end-tidal carbon dioxide was as low as 28 mmHg. The bronchoscopy revealed that endotracheal intubation was performed correctly, but the trachea was compressed and it was difficult to observe the right main bronchial inlet (Fig. 1d). Therefore, the endotracheal tube was exchanged with a 35-Fr double-lumen tube (Broncho-Cath™, Medtronic Japan, Tokyo, Japan) for one-lung ventilation of the left lung (Fig. 2a, b). Oxygenation was still poor; the SpO2 was 95% on a fraction of inspiratory oxygen (FiO2) of 80%. We decided to administer CT-guided puncture and drainage of the cyst contents to relieve the airway obstruction. When punctured, a dark brown fluid gushed out and a 7-Fr pigtail catheter (Hanako Drainage Kit™, Hanako Medical, Saitama, Japan) was placed inside the cyst. A total of 300 mL of fluid was aspirated, and stenosis of the airway disappeared (Fig. 2c, d). We confirmed that there was no bleeding or pneumothorax on the CT scan.

**Fig. 3:**
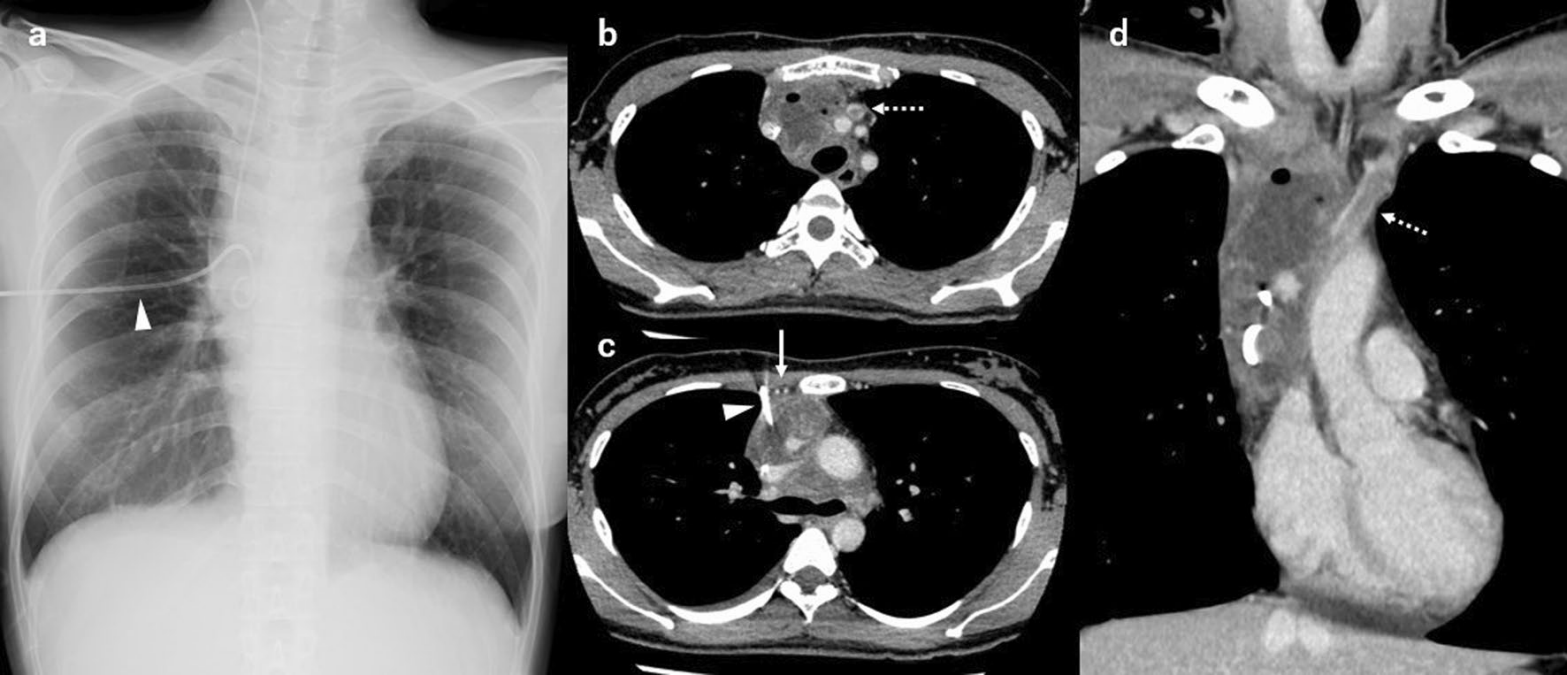
Chest X-ray image (**a**) and contrast-enhanced CT findings (**b**–**d**) after extubation. The image b is at the trachea level. The image c is at the level of carina of the trachea. The image d is the coronal section. The pigtail catheter (white arrowhead) was inserted laterally into the right internal thoracic artery and vein (white arrow). The left brachiocephalic vein (dotted white arrow) was surrounded by the tumor and thrombosed

After drainage, oxygenation improved. The arterial blood gas showed an FiO_2_ of 30%, a PaO_2_ of 149 mmHg, and a PaCO_2_ of 43 mmHg. Her blood pressure was 113/63 mmHg and her heart rate was regular (61/min). The endotracheal tube was extubated the next day and the pigtail catheter was left inside the tumor (Fig. 3a). Her symptoms of chest pain and dyspnea disappeared. The contrast-enhanced CT scan showed that the tumor had shrunk to a size of 8.8 × 5.0 × 4.8 cm (Fig. 3b–d). The left brachiocephalic vein was thrombosed and tumor invasion was suspected. Laboratory data of the effluent showed that the amylase level was markedly high at 5776 U/L. Cytological examination revealed no malignancy.

**Fig. 4:**
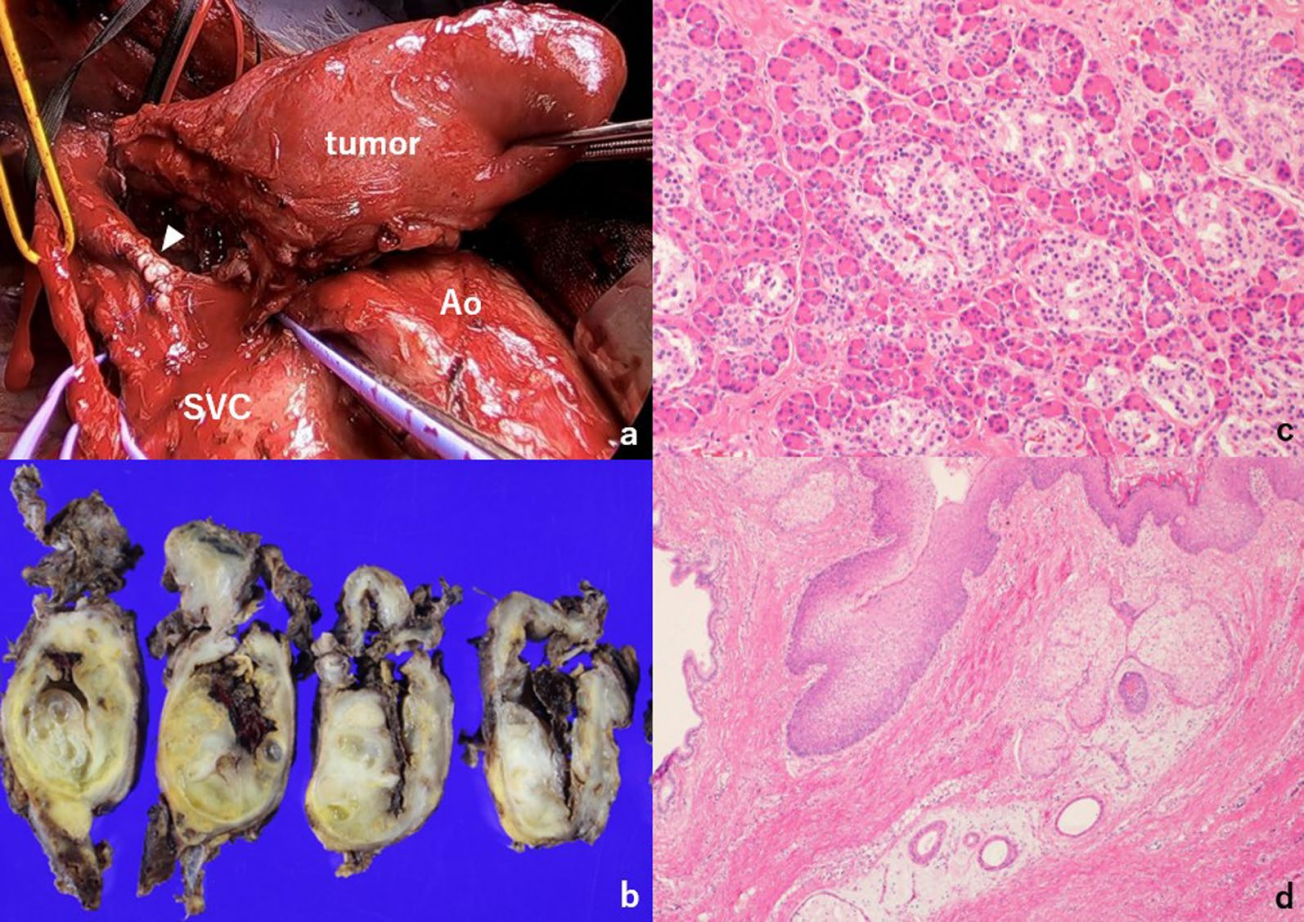
Intraoperative (**a**) and histopathological findings (**b**–**d**). **a** The tumor was adherent to the left brachiocephalic vein and trachea. The brachiocephalic vein was transected just before the junction of the right brachiocephalic vein (white arrow). **b** Macroscopic examination shows a mixture of cystic and solid components in the tumor. **c** Microscopic findings show the exocrine and endocrine glands of the pancreas (hematoxylin–eosin staining, high-power field). **d** Skin and skin appendages in the microscopic examination (hematoxylin–eosin staining, low-power field)

Surgery was performed 3 days after CT-guided drainage. General anesthesia was induced uneventfully, and median sternotomy was performed. The tumor was adherent to the left brachiocephalic vein and trachea, but not the lung. The left brachiocephalic vein was transected just before the junction of the right brachiocephalic vein (Fig. 4a). The pericardium was incised and there was no invasion of the superior vena cava or ascending aorta. Complete resection was performed; the operation time was 5 h and 45 min. The tumor was finally diagnosed as a mature teratoma (Fig. 4b–d). The patient was discharged on postoperative day 8 with no complications.

## Discussion

A giant mediastinal tumor may cause severe airway obstruction owing to its mass effects. In such cases, tracheal intubation following ventilator assistance does not provide effective respiratory support because it may be difficult to inhale tidal volume by pressure control or volume control against the bronchial obstruction caused by mechanical compression owing to the huge mass. General anesthetics and muscle relaxants decrease chest wall muscle tone, elevate the diaphragm, change the position of the tumor, and increase the risk of pressure obstruction of the airways and great blood vessels [1]. Furthermore, increased intrathoracic pressure due to positive pressure ventilation after intubation may worsen airway obstruction, resulting in further respiratory failure and circulatory collapse [2, 3]. In such cases, extracorporeal membrane oxygenation (ECMO) should be provided in conjunction with general anesthesia [4, 5]. In our case, CT-guided drainage of the fluid contents of the tumor was effective in decompressing the airway obstruction and allowed safe general anesthesia induction and surgical resection without ECLS. Prior to CT-guided drainage, the patient was assisted by a ventilator using a double-lumen tracheal tube to ensure at least one-lung ventilation without ECMO. The drainage prior to surgery also enabled a good surgical field to be achieved and ensured the detachment of the adhesions.

Preoperative ultrasound-guided drainage of giant mediastinal mature teratomas has been reported to prevent respiratory failure and circulatory collapse during the induction of general anesthesia [2, 6]. The advantage of ultrasound-guided drainage is that it is easy to perform at bedside in real-time without irradiation. However, lesions near the bone or lungs or deep lesions cannot be detected owing to artifacts and ultrasound attenuation [7]. CT-guided drainage enables the accurate assessment of the tumor, vascular location, puncture route, and depth from the skin. It is chosen for difficult-to-access or trans-ultrasound invisible lesions. Iatrogenic bleeding, such as hemothorax, pulmonary hemorrhage, or pneumothorax, may occur during image-guided drainage [8]. In our case, there was no iatrogenic complication during drainage. The pigtail catheter was inserted without injury to the right internal thoracic artery and vein. The disadvantage of CT-guided puncture is that it is impossible to perform at bedside and it causes exposure to radiation.

If the tumor is a cystic lesion, not solid, and compresses the airways or great blood vessels, preoperative CT-guided puncture is effective. In cases where malignant germ-cell tumor or malignant lymphoma is suspected, drainage may cause tumor dissemination. In this case, as we estimated that the tumor was not malignant based on normal levels of tumor markers and CT images, we decided to perform preoperative tumor drainage. Mediastinal mature teratomas are benign, but when ruptured, digestive enzymes or infectious materials can spread into the mediastinum or the thoracic cavity [9]. Similar conditions could occur after puncture. This can cause mediastinitis or pleurisy and severe adhesion; thus, early surgical resection is needed after drainage.

## Conclusions

Rescue preoperative CT-guided drainage of a giant mediastinal mature teratoma allowed safe general anesthesia and operation without deterioration of the respiratory and circulatory systems by releasing airway obstruction.

## Data Availability

All data are presented within the manuscript.

## Abbreviations

CT: Computed tomography
ECLS: Extracorporeal life support
SpO_2_: Saturation of percutaneous oxygen
PaO_2_: Partial pressure of oxygen
PaCO_2_: Partial pressure of carbon dioxide
FiO_2_: Fraction of inspiratory oxygen
ECMO: Extracorporeal membrane oxygenation
